# The adhesion molecule Necl-3/SynCAM-2 localizes to myelinated axons, binds to oligodendrocytes and promotes cell adhesion

**DOI:** 10.1186/1471-2202-8-90

**Published:** 2007-10-29

**Authors:** François Pellissier, Alan Gerber, Christoph Bauer, Marc Ballivet, Vincent Ossipow

**Affiliations:** 1 Department of Biochemistry, University of Geneva, 30 Quai Ernest Ansermet, Sciences II, 1211 Geneva 4, Switzerland; 2 NCCR Frontiers in Genetics, Imaging Platform, University of Geneva, 30 Quai Ernest Ansermet, Sciences II, 1211 Geneva 4, Switzerland

## Abstract

**Background:**

Cell adhesion molecules are plasma membrane proteins specialized in cell-cell recognition and adhesion. Two related adhesion molecules, Necl-1 and Necl-2/SynCAM, were recently described and shown to fulfill important functions in the central nervous system. The purpose of the work was to investigate the distribution, and the properties of Necl-3/SynCAM-2, a previously uncharacterized member of the Necl family with which it shares a conserved modular organization and extensive sequence homology.

**Results:**

We show that Necl-3/SynCAM-2 is a plasma membrane protein that accumulates in several tissues, including those of the central and peripheral nervous system. There, Necl-3/SynCAM-2 is expressed in ependymal cells and in myelinated axons, and sits at the interface between the axon shaft and the myelin sheath. Several independent assays demonstrate that Necl-3/SynCAM-2 functionally and selectively interacts with oligodendrocytes. We finally prove that Necl-3/SynCAM-2 is a *bona fide *adhesion molecule that engages in homo- and heterophilic interactions with the other Necl family members, leading to cell aggregation.

**Conclusion:**

Collectively, our manuscripts and the works on Necl-1 and SynCAM/Necl-2 reveal a complex set of interactions engaged in by the Necl proteins in the nervous system. Our work also support the notion that the family of Necl proteins fulfils key adhesion and recognition functions in the nervous system, in particular between different cell types.

## Background

Multicellular organization entails cell-cell recognition and adhesion. The cell adhesion molecules (CAMs) are among the specialized plasma membrane proteins that carry out these functions. The mechanisms of recognition and adhesion are of particular relevance in the nervous system whose operation heavily relies on cell-cell communication, and whose many cell types acting in concert are capable of extensive re-organization in development, learning and memory. Recently two related CAMs, Necl-2-SynCAM [1-4] and Necl-1 [5], were shown to fulfill important functions in the central nervous system (CNS). In addition to acting as a CAM in other tissues [6-11], SynCAM can induce presynaptic differentiation in co-cultured neurons [1,4], whereas Necl-1 is expressed specifically in brain and localizes at contact sites between neurons and glial cells [5]. These two CAMs are Ig superfamily members and genomic analysis predicts that they are part of a set of four closely related proteins [1,12-15] for which different nomenclatures have been proposed, in particular nectin-like 1 to 4 (Necl-1 to -4), and synaptic CAM 1 to 4 (SynCAM-1 to -4), each with its merits [1,13,15,16].

Here we describe Necl-3/SynCAM-2, a previously uncharacterized member of the family, which we term Necl-3 throughout for simplicity and because the term is neutral with respect to function. Necl-3 shares with the other Necls/SynCAMs a conserved modular organization comprising three Ig domains, a single trans-membrane pass and a short cytoplasmic region containing 4.1 and PDZ binding motifs [1,12-15]. Necl-3 accumulates in several tissues, including those of the nervous system, where it localizes to myelinated axons and in ependymal cells. We also demonstrate that Necl-3 engages in homo- and heterophilic interactions leading to cell aggregation and discuss its possible implication in processes dependent on neural cell adhesion.

## Results

### Necl-3 expression

**Figure 1:**
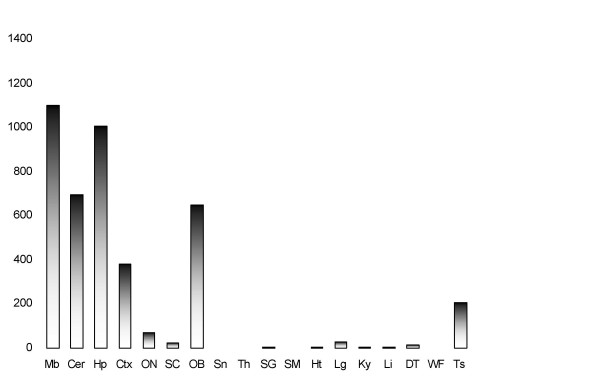
All signal were normalized with GAPDH and are displayed as arbitrary units of the fluorescence intensity (Mb: midbrain, Cer: cerebellum, Hp: hippocampus, Ctx: cortex, ON: optic nerve, SC: spinal cord, OB: olfactory bulb, Sn: snout, Th: thymus, SG: surrenal gland, SM: skeletal muscle, Ht: heart, Lg: lung, Ky: kidney, Li: liver, DT: digestive tract, WF: white fat, Ts: testis.

**Figure 2:**
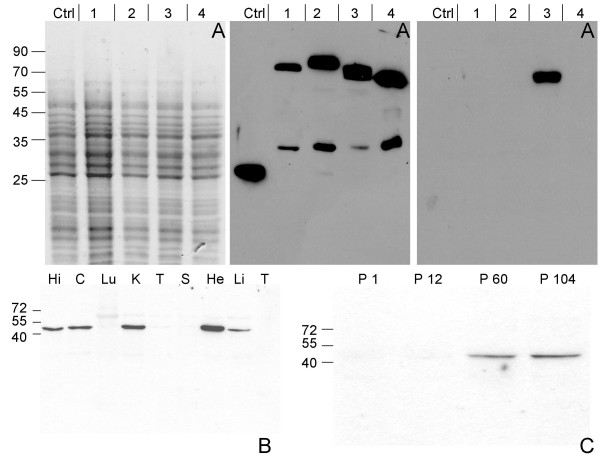
Antibody specificity and Necl-3 expression. (Panel A) Crude lysates of S2 cells transfected with either GFP or one each of the Necl-GFP fusion proteins were visualized by SDS-PAGE and coomassie blue staining (left panel). The gels were blotted on nitrocellulose membranes and revealed with either an anti GFP mAb (middle panel), or the affinity-purified anti Necl-3 antibody (right panel). The cells had been transfected with GFP (Ctrl), Necl-1-GFP (1), Necl-2-GFP (2), Necl-3-GFP (3) or Necl-4-GFP (4). The anti GFP mAb reveals fusion proteins of the expected apparent molecular weights as well as faster migrating products, likely the result of degradation or internal translation initiation. The anti Necl-3 antibody does not cross-react with the other members of the Necl family. (Panel B) 15 μg of homogenates from hippocampus (Hi), cortex (C), lung (Lu), kidney (K), testis (T), spleen (S), heart (He), liver (Li) and thymus (T) of a 2-month-old rat were subjected to SDS-PAGE, followed by western blotting with the anti-Necl-3 antibody. (Panel C) Rat cortex homogenates from the indicated postnatal ages were subjected to SDS-PAGE (12%), followed by western blotting with the anti-Necl-3 antibody.

Genes that are differentially expressed in the postnatal development of the rat CNS may inform on important aspects of neurogenesis [17]. One such gene codes for Necl-3, whose expression we assessed in various rat tissues by northern blot and real-time PCR. A Necl-3 mRNA greater than 5 Kb was detected in various structures of the CNS (midbrain, cerebellum and hippocampus), whereas it was either undetectable or poorly expressed in all other organs tested except for testis (data not shown). Real-time PCR analysis of eighteen tissues using a primer pair spanning two exons confirmed the northern blot result (Fig. 1). We next examined Necl-3 protein accumulation. Since there is a high degree of homology among the Necl family members (in the rat, Necl-3 has 48%, 44%, and 35% amino-acid identity to Necl-1, Necl-2, and Necl-4 respectively), it was critical to ensure specificity when raising and testing anti Necl-3 antibodies. We immunized rabbits against a recombinant segment of the extracellular domain of Necl-3 that is the least conserved region among the Necl proteins. Antibody specificity was tested using *Drosophila *S2 cells transfected with either green fluorescent protein (GFP) alone or Necl-1, Necl-2, Necl-3, and Necl-4 fused to GFP at their carboxy-termini. Crude S2 lysates were separated by SDS gel electrophoresis and probed with either the anti Necl-3 or an anti GFP antibody serving as a loading control (Fig. 2A). The anti Necl-3 antibody reveals a single species of the expected size exclusively in cells transfected with Necl-3-GFP. We conclude that we have a highly specific anti Necl-3 antibody preparation that does not cross-react with Necl-3's closest relatives, i.e. Necl-1, Necl-2, or Necl-4.

Using the Necl-3 antibody, we ran western blots on extracts from a series of different organs. The Necl-3 protein was detected in brain structures, but also in kidney, heart and liver (Fig. 2B). Necl-3 migrates with an apparent mass of approximately 52 kDa, which is slightly more than its calculated molecular weight of 48 kDa. This is due at least in part to N-linked glycosylation since treatment with the N-glycosidase PNGaseF converts Necl-3 to a faster migrating form, consistent with the presence of N-glycosylation consensus sites in the extracellular domain (data not shown). We also monitored the accumulation of Necl-3 in the postnatal brain and found that the protein is barely detectable at P1 or P12 and has reached a plateau by P60 (Fig. 2C).

### Necl-3 is targeted to the plasma membrane and engages in homo- and heterophilic interactions

**Figure 3:**
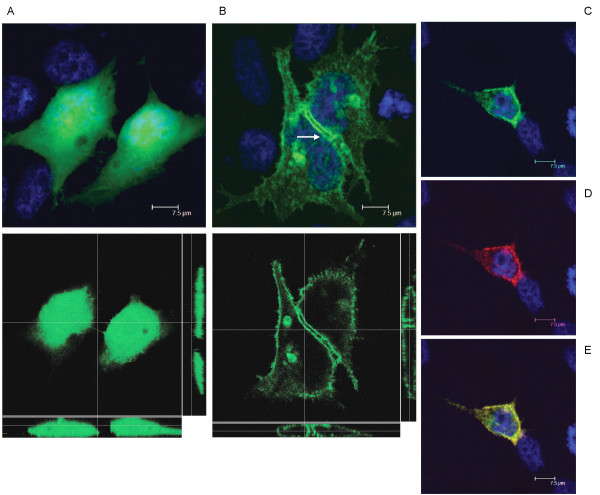
GFP (A, top panel: average projection of 30 z-stacks of 0.24 micron each; bottom panel: a single X/Y plane in the center, a X/Z slice at bottom and a Y/Z slice on the right) or Necl-3-GFP (B, top panel: average projection of 39 z-stacks of 0.12 micron each; bottom panel: a single X/Y plane in the center, a X/Z slice at bottom and a Y/Z slice on the right) was transfected in Hela cells and visualized by confocal fluorescence microscopy. Unlike GFP alone, Necl-3 accumulates in the plasma membrane and at cell-cell contacts between transfected cells (arrow). (C-E), Hela cells transfected with Necl-3-GFP were processed for immunofluorescence with the anti Necl-3 antibody in non-permeabilized conditions and visualized (average projection of 83 z-stacks of 0.12 micron each) in the GFP channel (C) and in the rhodamine channel (D). To reveal untransfected cells, the specimen was stained with DAPI. The (E) panel shows the merged DAPI, GFP and rhodamine channels. Note that the Necl-3 antibody only stains the transfected cells.

**Figure 4:**
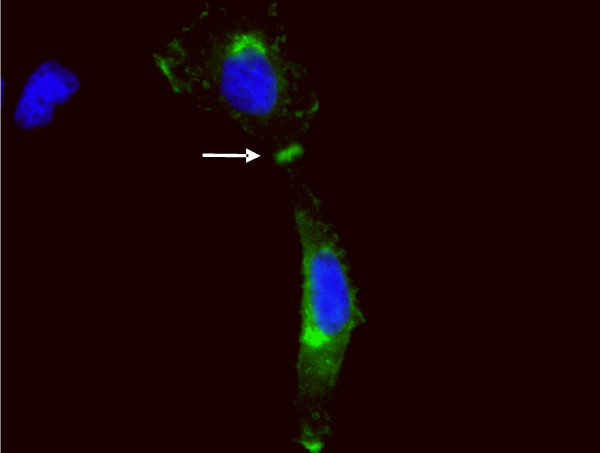
Myc-tagged Necl-3 was transfected in Hela cells. Anti myc mAb was applied and revealed with a secondary antibody coupled to FITC. Necl-3-myc accumulates at cell contacts (arrow) as does a GFP-tagged Necl-3.

Necl-1 and Necl-2 form homo- and heterophilic interactions [1,8-10,18]. In order to investigate Necl-3's potential to insert in the plasma membrane, we transfected Hela cells with vectors expressing either GFP or a chimeric protein consisting of Necl-3 carboxy-terminally fused to GFP. Unlike GFP alone (Fig. 3A), Necl-3-GFP accumulates at contact sites between transfected cells (Fig. 3B). Confocal analysis and tri-dimensional reconstitution confirm the cytoplasmic location of GFP and the plasma membrane location of Necl-3-GFP (Fig. 3A, B). To rule out a localization artifact due to the GFP moiety of the fusion protein, we transfected a myc-tagged version of Necl-3, and identical results were observed (Fig. 4). These data imply that Necl-3 is targeted to the plasma membrane and possibly engages in homophilic trans-interactions. Similar results were obtained for Necl-1 and Necl-2 [5,10,19,20]. To verify that Necl-3 is targeted to the plasma membrane, we took advantage of the fact that our anti Necl-3 antibody recognizes a domain predicted to be extracellular. Transfected Hela cells were used in immunofluorescence experiments done on cells that were not permeabilized, i.e. in the absence of detergent. In such conditions the antibody is indeed able to specifically bind to the putative extracellular domain of Necl-3 (Fig. 3C–E), confirming its predicted topology and location at the plasma membrane.

**Figure 5:**
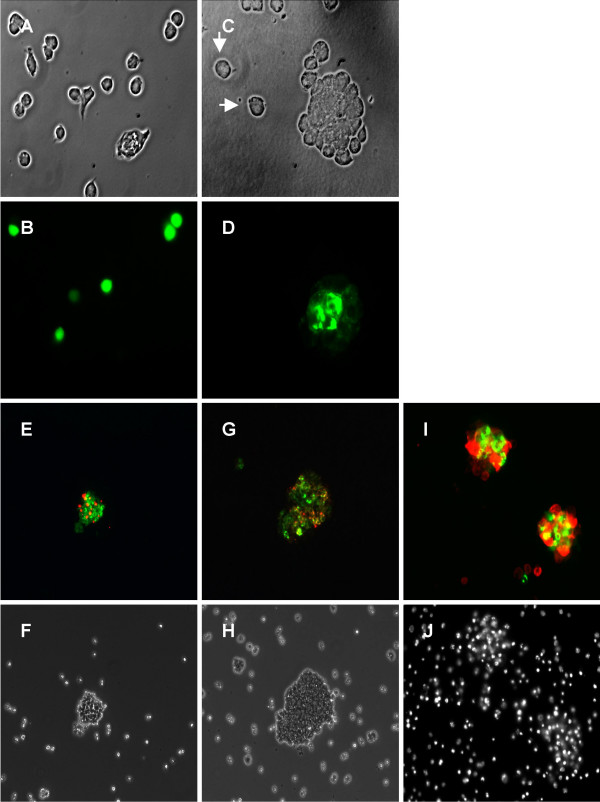
S2 cells transfected with GFP (A, B) or Necl-3-GFP (C, D) were gently shaken and visualized by phase contrast (A, C) and fluorescence microscopy (B, D). Cell aggregates form with Necl-3-GFP transfected cells (C, D), whereas GFP transfected (A, B) and untransfected cells (arrows in C) do no aggregate. S2 cells transfected with Necl-3-GFP were mixed with Necl-1-DsRed cells (E, F); cells transfected with Necl-3-GFP were mixed with Necl-4-DsRed (G, H) cells, and cells transfected with Necl-3-myc were mixed with Necl-2-GFP (I, J). The cell aggregates were visualized by fluorescence microscopy with green and red pass filters (E, G, I), by phase contrast (F, H), or by fluorescent staining of the nuclei with DAPI (J).

**Figure 6:**
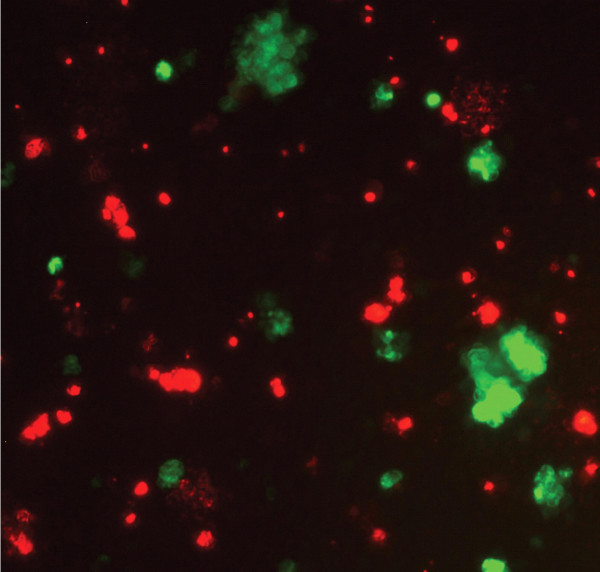
S2 cells transfected with ALCAM-GFP or Necl-3-DsRed were mixed, then gently shaken and visualized by fluorescence microscopy. Cells aggregate only homotypically, the red aggregates being mediated by Necl-3-Ds-Red and the green ones by ALCAM-GFP.

In order to further characterize the homo- and heteromerizing activities of Necl-3, we performed aggregation assays in S2 cells. These do not aggregate spontaneously and Necl-2/SynCAM is able to induce their aggregation through homophilic interaction [1]. S2 cells were transfected separately with plasmids expressing GFP or Necl-3-GFP under the control of an inducible metallothionein promoter. After transfection, the cells were washed free from plasmid, induced with CuSO_4_, gently shaken to favor aggregation and visualized without further fixation. Unlike cells transfected with GFP (Fig. 5A, B), those transfected with Necl-3-GFP form large aggregates (Fig. 5C–D). Since the transfection efficiency is not 100%, the non-transfected cells in the Necl-3-GFP plate serve as internal control, showing no aggregation (Fig. 5C, D). We next tested Necl-3's ability to form aggregates with its relatives Necl-1, Necl-2 and Necl-4. S2 cells were separately transfected with plasmids coding for Necl-3-GFP, Necl-1-DsRed, and Necl-4-DsRed. After transfection, they were mixed in different combinations pairwise and induced to express their transfected Necl. After gentle shaking, aggregates formed between Necl-3-GFP and Necl-1-DsRed (Fig. 5E, F) or Necl-4-DsRed (Fig. 5G, H). Again, non-transfected cells serving as internal control did not aggregate. To rule out an artifact due to the use of DsRed, we performed a similar S2 cells aggregation assay using a myc-tagged version of Necl-3 along with Necl-2-GFP. Using a rhodamine-conjugated anti myc secondary antibody, we again observed aggregates of red and green cells (Fig. 5I, J). Thus, Necl-3 unambiguously interacts with itself, Necl-1, Necl-2, and Necl-4 in these aggregation assays. To test the promiscuity of Necl-3's heterophilic interactions, we assayed its ability to aggregate with ALCAM/CD166, an adhesion molecule bearing five Ig-loops [21,22]. Necl-3-DsRed and ALCAM-GFP were used for aggregation assays as described above. Large S2 cells aggregates were observed but these were always composed of green only or red only cells (Fig. 6). The fact that Necl-3 does not interact productively with ALCAM shows that, even though Necl-3 is capable of multiple heterophilic interactions with other Necl proteins, it retains specificity for certain Ig loop folds.

**Figure 7:**
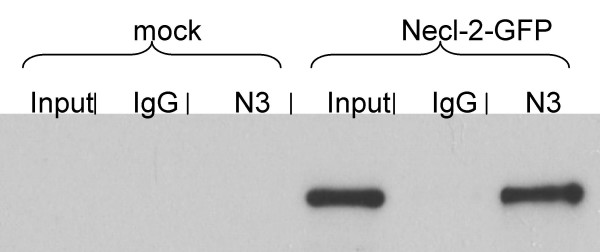
The Fc-Necl-3 fusion protein (N3) or IgGs (IgG) were immobilized on protein A beads. These were used for pull-downs in lysates of S2 cells transfected either with a control vector (mock) or with Necl-2-GFP. After the pull-downs, the proteins retained on the beads were subjected to SDS-PAGE (12%), followed by western blotting with an anti GFP mAb. 15% of the input lysate material was loaded on the same gel (input).

In order to obtain a biochemical confirmation that Necl-3 is able to make heterophilic interactions, a fusion protein between the Fc domain of a human IgG and the extra-cellular domain of Necl-3 was produced (Fc-Necl-3). This chimeric protein or a control full-length human IgG were adsorbed on protein A resin and used in pull-down assays. Lysates of mock-transfected and Necl-2-GFP transfected S2 cells were the inputs for the pull-down experiments. After incubation and washings, the proteins retained on the beads were analyzed by western blot (Fig. 7). Control IgG beads are unable to bring down Necl-2-GFP, whereas the Fc-Necl-3 beads efficiently do so (approximately 15% of the input Necl-2-GFP is recovered). Incidentally, the fact that human IgG beads do not interact with Necl-2 confirms that even though the Necl proteins are capable of multiple heterophilic interactions, they retain specificity. We note that the heterophilic interaction described here is calcium independent since the pull-down experiments were done in presence of EDTA. Similar data have been reported for the homophilic interactions of Necl-2 [1].

### The distribution of Necl-3 in the nervous system

**Figure 8:**
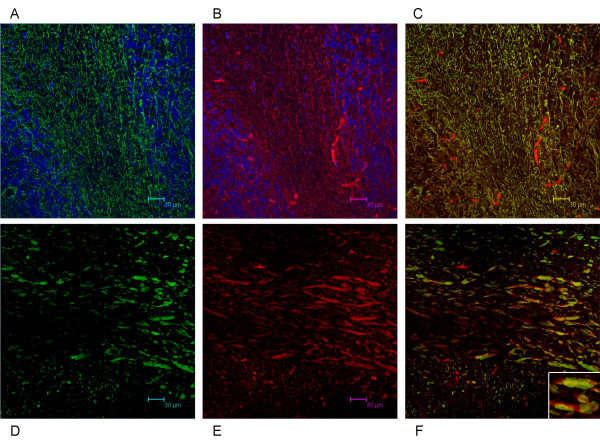
12 μm sagittal brain sections of a two-month-old rat were processed for immunohistochemistry. The top sections (A, B, C) show white matter and granula within a cerebellar fold (average projection of 15 z-stacks of 0.16 micron each). The DAPI stained nuclei appear in blue and reveal the nuclei-dense granula regions bracketing the white matter. The bottom sections (D, E, F) show the ventral reticular nucleus (average projection of 9 z-stacks of 0.16 micron each). The sections were incubated with anti-Necl-3 and anti neurofilament antibodies, which were revealed with secondary antibodies coupled to Alexa 488 and Alexa 594, respectively. Panels (A) and (D) show neurofilament immunoreactivity in the green-pass channel, and panels (B) and (E) Necl-3 immunoreactivity in the red-pass channel. The green and red images are merged in (C) and (F). Nuclei were visualized with DAPI in (A) and (B). The inset of Figure 5F shows that Necl-3 and neurofilament do not perfectly overlap.

**Figure 9:**
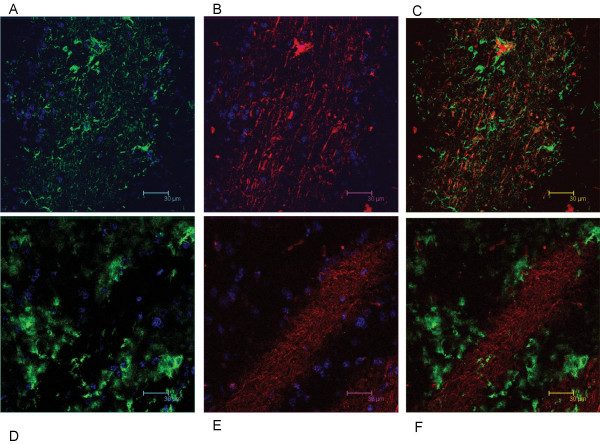
12 μm sagittal sections of the caudate putamen in a two-month-old rat were processed for immunohistochemistry. The sections were incubated with anti Necl-3 and anti GFAP antibodies (A, B, C, 14 z-stacks of 0.12 micron each) or with anti Necl-3 and anti CD11b antibodies (D, E, F, 20 z-stacks of 0.36 micron each), which were revealed with secondary antibodies as in Fig. 5. (A), GFAP immunoreactivity in the green-pass channel; (B), Necl-3 immunoreactivity in the red-pass channel; (D), CD11b immunoreactivity in the green-pass channel; (E), Necl-3 immunoreactivity in the red-pass channel. The green and red images are merged in (C) and (F). Nuclei were visualized with DAPI in (A), (B), (D), and (E).

**Figure 10:**
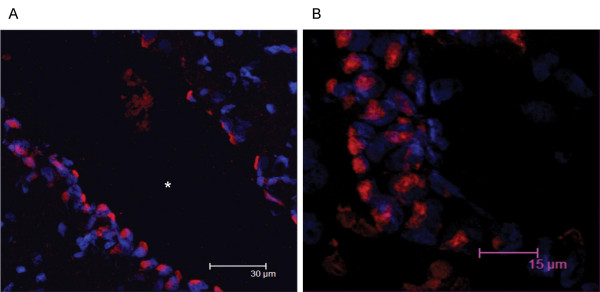
12 μm sagittal brain sections of a two-month-old rat were processed for immunohistochemistry. The sections were incubated with anti-Necl-3 antibody, which was revealed with a secondary antibody coupled to Alexa 594. Nuclei were visualized with DAPI. Panel A (average projection of 64 z-stacks of 0.12 micron each) shows part of the third ventricle (asterisk) bordered by the ependymal cell layer. Panel B (average projection of 57 z-stacks of 0.12 micron each) shows ependymal cells of the choroid plexus in the third ventricle.

We have shown that Necl-3 transcript (Fig. 1) and protein (Fig. 2B, C) are strongly expressed in the CNS. Closely-related Necl-2 is also expressed there [2,3], where it can induce presynaptic differentiation in co-cultured neurons [1,4], while Necl-1 localizes at cell-cell contact sites between neurons and glial cells [5]. We therefore investigated the brain distribution of Necl-3 by immunohistochemistry and confocal microscopy. Intense labeling was observed in structures resembling axon bundles in several brain areas including cerebellar white matter and ventral reticular nucleus(Fig. 8A, D). The axonal localization of Necl-3 was confirmed by a near-perfect overlap with the axonal neurofilament marker (Fig. 8A–F). Careful examination however shows that although the Necl-3 and the neurofilament staining are largely overlapping, they are not identical (inset Fig. 8F). This co-localization is unlikely to be an artifact since it was apparent for each Z optical section examined (not shown). We investigated whether Necl-3 is expressed in other cell types in co-labeling experiments with non-neuronal markers. Antibodies against GFAP (glial fibrillary acid protein) revealed very little or no accumulation in astrocytes and anti CD11b showed no detectable signal in microglial cells (Fig. 9A–F). Necl-3 immunoreactivity was also detected in ependymal cells of the ventricle wall and choroid plexus (Fig. 10A–B). Close examination shows that in addition to its apparent membrane localization, Necl-3 also accumulates to the cytoplasm of ependymal cells, the significance of which is currently under investigation. Finally, labeling was observed inside capillaries, indicating that Necl-3 is also expressed on cells circulating in the blood (data not shown).

**Figure 11:**
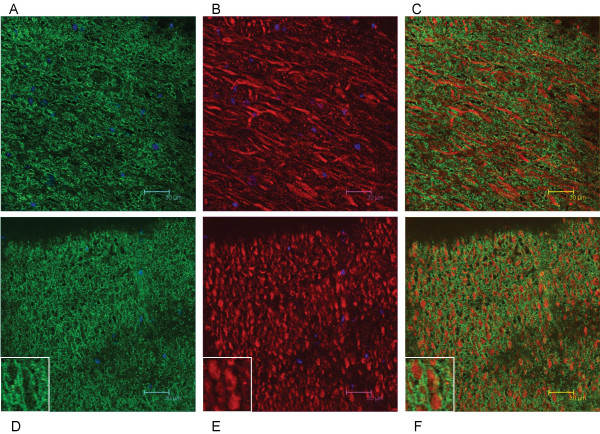
12 μm sagittal brain sections of a two-month-old rat were processed for immunohistochemistry. The vestibular nucleus is visualized in the top sections (A, B, C) as the average projection of 20 z-stacks of 0.24 micron each, and in the bottom sections the pons (D, E, F) as the average projection of 26 z-stacks of 0.12 micron each. The sections were incubated with anti-Necl-3 and anti Rip antibodies (A, B, C) or with anti-Necl-3 and anti MBP antibodies (D, E, F), which were revealed with secondary antibodies as in Fig. 5. (A), Rip immunoreactivity in the green-pass channel; (B), Necl-3 immunoreactivity in the red-pass channel; (D), MBP immunoreactivity in the green-pass channel; (E), Necl-3 immunoreactivity in the red-pass channel. The green and red images are merged in (C) and (F). Nuclei were visualized with DAPI in (A), (B), (D), and (E).

**Figure 12:**
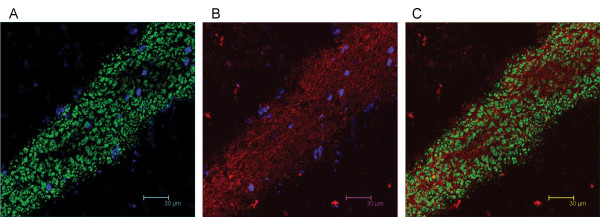
12 μm sagittal sections of the caudate putamen in a two-month-old rat were processed for immunohistochemistry. The sections were incubated with anti-Necl-3 and anti Rip antibodies, which were revealed with secondary antibodies as in Fig. 5. (A), Rip immunoreactivity in the green-pass channel; (B), Necl-3 immunoreactivity in the red-pass channel. The green and red images are merged in (C). Nuclei were visualized with DAPI in (A) and (B). All images represent the average projection of 9 z-stacks of 0.12 micron each. Note that although present in the same areas, the Necl-3 and oligodendrocytic immunoreactivities are not overlapping.

**Figure 13:**
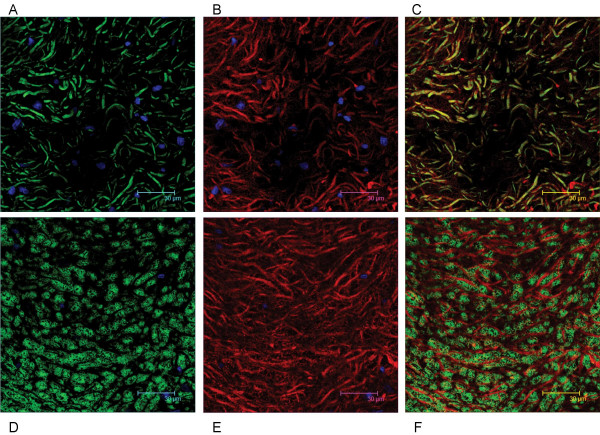
12 μm trigeminal nerve sections of a four-month-old rat were processed for immunohistochemistry. The sections were incubated with anti-Necl-3 and anti neurofilament antibodies (A, B, C, 10 z-stacks of 0.24 micron each) or with anti-Necl-3 and anti MBP antibodies (D, E, F, 10 z-stacks of 0.12 micron each), which were revealed with secondary antibodies as in Fig. 5. (A), neurofilament immunoreactivity in the green-pass channel; (B), Necl-3 immunoreactivity in the red-pass channel; panel (D), Rip immunoreactivity in the green-pass channel; (E), Necl-3 immunoreactivity in the red-pass channel. The green and red images are merged in (C) and (F). Nuclei were visualized with DAPI in (A), (B), (D), and (E).

**Figure 14:**
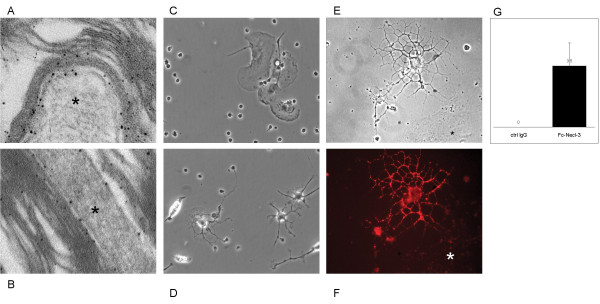
Panels (A and B) show examples of immuno-electron microscopy with anti Necl-3 antibody in the pons of three-month old rats. The signal was enhanced with nanogold particles. Note the immunoreactivity at the interface between the axon shaft (star) and the concentric myelin folds. The myelin sheaths locally detach from the axon due to the fixation and embedding procedures. Panel (C and D) shows representative fields of types of cells attached to the control IgGs (C) or to Fc-Necl-3 treated plates (D). Panel (E and F), Fc-Necl-3 bound to an oligodendrocyte. The phase contrast picture shows a typical oligodendrocyte as well as another cell (*), probably an astrocyte (Panel E). The image in the red-pass channel shows that, unlike the astrocyte (*), the oligodendrocyte binds to Fc-Necl-3, as revealed by a rhodamine-coupled secondary antibody (Panel F). Panel (G) The numbers of oligodendrocytes per field (Y-axis) were scored in control dishes (ctrl Ig) and Fc-Necl-3 coated dishes (error bars represent 1 standard deviation, P(T≤t) = 0.018)

To further characterize Necl-3's axonal location, co-labeling experiments were performed with the Rip mAb that specifically recognizes the oligodendrocytic myelin protein CNP (2',3'-cyclic nucleotide 3'-phosphodiesterase) [23,24]. In sharp contrast to the co-localization seen with neurofilament, Necl-3 positive fibers are always surrounded by Rip/CNP immunoreactivity in a mutually exclusive pattern (Fig. 11A–C). Identical results were obtained using a mAb directed against the myelin basic protein (MBP) in combination with anti Necl-3 antibody (Fig. 11D–F). This confirms that Necl-3 is found in the axoplasm of myelinated axons. We also investigated the relationship between Necl-3 and myelination by examining the caudate putamen stained with anti Necl-3 and anti Rip/CNP antibodies. There we found that Necl-3 immunoreactivity is stronger in the myelinated areas and, as seen previously, that it is wrapped in oligodendrocytic immunoreactivity (Fig. 12A–C). We finally examined whether Necl-3 is also expressed in myelinated axon fibers of the PNS. Co-labeling experiments with anti Necl-3 antibody plus anti neurofilament antibody (Fig. 13A–C) or Rip/CNP antibody (Fig. 13D–F) show that in the trigeminal nerve, as in the CNS, Necl-3 immunoreactivity overlaps perfectly with that of the neurofilaments and is surrounded by but excluded from the myelinated processes. Electron microscopy was used to confirm at a finer scale that Necl-3 is present at the axon-oligodendrocyte interface. The pons of three-month-old rats was used for pre-embedding immuno-electron microscopy with affinity-purified anti Necl-3 antibody. These experiments show that Necl-3 is absent from the axonal matrix and largely, but not exclusively, located at the innermost interface between oligodendrocytic layers and the axon shaft (Fig. 14A and 14B). Control experiments with an antibody directed against the extra-cellular domain of the related Necl-4 protein fail to show the same distribution (data not shown).

### Necl-3 selectively binds to oligodendrocytes

**Figure 15:**
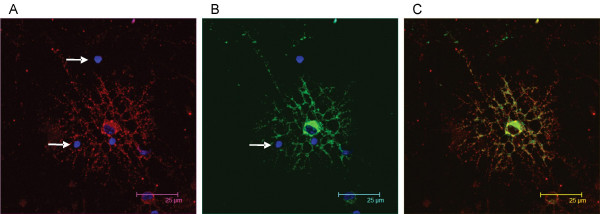
(A) The image in the red-pass channel shows Fc-Necl-3 revealed by a rhodamine-coupled secondary antibody. (B) The green-pass channel shows that Fc-Necl-3 binds to oligodendrocytes, which are identified by the specific mAb RIP revealed with an Alexa 594 secondary antibody. Note that Rip-negative cell-types are not bound by Fc-Necl-3 (arrows in A and B). The green and red images are merged in (C). Nuclei were visualized with DAPI in (A), (B). The field shown in the figure is representative of more than 100 rhodamine-positive cells, all of which were also Rip-positive.

During CNS development and plasticity, countless different interactions can form between various cell types and subtypes. To explore whether Necl-3 would preferentially form contacts with specific cells we relied on an adhesion assay with mixed primary cultures [25]. Cerebellar cells were dissociated and plated on petri dishes that were either untreated, coated with control IgGs, or coated with Fc-Necl-3. After a short incubation time, the non-adhering cells were washed away, and the remaining cells were cultured for another day. Microglial cells attached non-specifically to all dishes, reflecting the ability of these cells for binding untreated surfaces. Only the Fc-Necl-3 treated dishes attached cells other than microglia, and these were exclusively oligodendrocytes, as evident from their characteristic morphology (Fig. 14C and 14D) and immunoreactivity for Rip/CNP (data not shown). Quantification indicates that this result is statistically highly significant (Fig. 14G). Thus in this system Necl-3 can functionally and selectively interact with oligodendrocytes and does not bind to other CNS cell-types. To confirm the observation that Necl-3 preferentially binds to oligodendrocytes, the fusion protein Fc-Necl-3 or control IgGs were directly added to a mixed primary culture prepared from neonatal cortex. The Fc moiety on control IgGs and Fc-Necl-3 was revealed with labeled anti human Fc antibodies. No signal was observed with the control IgGs by fluorescent microscopy (data not shown), in contrast oligodendrocytes were strongly and selectively labeled with Fc-Necl-3 (Fig. 14E and 14F). The binding of Fc-Necl-3 on mixed primary cultures was repeated and the oligodendrocytes were revealed with the mAb Rip that specifically stains these cells. Again oligodendrocytes are preferably bound by Necl-3, despite the presence of other cell types in the cultures (Fig. 15), and incubating the cultures with the same amount of IgGs does not to produce any detectable signal (data not shown). These experiments are in complete agreement with the plate adhesion assay and indicate that Necl-3 has a ligand on oligodendrocytes, to which it preferentially binds. This is also in agreement with observation by others that in the CNS, Fc-Necl-3 binds to oligodendrocytes [26].

## Discussion

Several laboratories have predicted the existence of Necl-3, based on bioinformatic considerations [1,12-15]. Biederer has done a particularly interesting analysis with respect to splice variants, gene size, and protein structure [15]. Here we describe several of Necl-3's biological features. We demonstrate that it is a *bona fide *adhesion molecule able to engage in homo- and heterophilic interactions. The contacts Necl-3 makes with itself and with Necl-1, -2 and -4 are strong enough to withstand co-precipitation and cause cell aggregation. Likewise, Kakunaga et al. have shown that Necl-1 interacts with itself, Necl-2, nectin-1 and nectin-3 [5], whereas Necl-2/SynCAM binds to itself and to Necl-1, nectin-3 and CRTAM [1,8-10,12,20,27,28]. It was also recently reported that Necl-1 and Necl-4 binds homo- and heterophilically and that Necl-2 and Necl-3 bind to Necl-1 and Necl-4 [26]. In this latter report, the authors use a different technology than ourselves and reach similar conclusions, i.e. that Necl proteins engage in homo- and heterophilc interaction. The degree of promiscuity of the Necl proteins is an important biological question. It is unlikely that they only interact with the partners so far documented, but do they interact with many other Ig domain proteins and with proteins of other superfamilies? Here we show that Necl-3 does not react in trans with the Ig folds in IgG's and in ALCAM, but much more work will be needed to sort out the range of these interactions and clarify their biological relevance.

Using highly specific antibodies, we show that Necl-3 is enriched in myelinated axons in rat CNS and PNS. Our immuno-electron microscopy data indicate that Necl-3 accumulates at contact sites between neurons and oligodendrocytes. In addition, initial investigations suggest that Necl-3, similarly to Necl-1 [5,26], is located at internodes on myelinated axons (data not shown). A function of Necl-3 compatible with these data, as well as with the fact that its maximal expression coincides with mature myelination, would be that Necl-3 participates in the myelin ensheathment of the axon [29,30]. It could do so by "gluing" the oligodendrocytic processes to the axon shaft, a proposition that should be investigated in the future. Our adhesion assays show that Necl-3 preferentially adheres to oligodendrocytes. Since it is mostly expressed in neurons, this suggests that although capable of forming homophilic trans-interactions, Necl-3 preferentially forms heterophilic interactions. It raises the question of the identity of the Necl-3 interaction partner(s) in the oligodendrocyte. A good candidate for such a partner is Necl-1, as it is present in oligodendrocytes [5,31] and binds to Necl-3 (this work). A recent report showed very convincingly that in the PNS Necl-1 is axonal and segregates preferentially to the internodal membrane whereas Necl-4 is expressed in Schwann cells and heterodimerizes with Necl-1, thereby forming a pair mediating axon-glia contact [26]. Necl-4 is therefore a likely partner for Necl-3 in the CNS, as these two Necl proteins are able to make heterophilic interactions (our work and [26]). Indeed Spiegel et al. also report, as data not shown, that in the CNS Fc-Necl-3 bind to oligodendrocytes [26]. Consequently, Necl-3 (this report and [26]) and Necl-1 [5,26] are likely involved in the establishment axon-glia contacts. However, numerous other oligodendrocytic proteins could also serve as plausible Necl-3 partners [31,32].

## Conclusion

The complex set of interactions engaged in by the Necl proteins suggests that they fulfill important cell adhesion and recognition functions. Our results reinforce the notion that the Necl family of proteins is involved in cell adhesion and recognition in the nervous system, as recently proposed [31]. There, Necl-1 and Necl-3 would favor neuron-glia contact (this work and [5]) through heterophilic binding to Necl-4 [26]; and Necl-2 would enable neuron-neuron contact [1,15]. Intriguingly, Necl-2 also promotes interactions between the nervous and immune systems [20,27,28,33]. It will be of interest to test whether other Necl family members fulfill similar neuro-immune functions. It also points to a possible mechanism for the interaction between lymphocytes and myelinated axons as it occurs in demyelinating diseases such as multiple sclerosis, leukodystrophy, central pontine myelinolysis and others. Here, Necl-2 on lymphocytes would interact with Necl-4 on oligodendrocytes or Necl-1, Necl-2, or Necl-3 on the axon shaft. The Necl proteins may also be involved in certain aspects of nervous system regeneration and repair. In a PNS remyelination model, Necl-4 appears to be important for this process [26], and Necl-2 (SgIGSF) mRNA and protein are upregulated in a model of olfactory epithelium nerve transection [34]. Since Necl-3 is not present exclusively in the nervous system, it will be important to assess its cellular localization in the other tissues that express it.

In conclusion, our data describe some of the properties of the novel adhesion molecule Necl-3 and highlight its implication in the nervous system. This work also supports the notion that the Necl family of proteins fulfills important adhesion and recognition functions in the nervous system.

## Methods

### Plasmid construction

PCR reactions were done with Pfu Turbo polymerase (Stratagene) according to the supplier's instructions. The mammalian expression vectors pNecl-3-GFP and pNecl3-DsRed2 were constructed as follows: the cDNA DKFZp761G128 (accession AL834270) was used as a template for PCR amplification with the primers 5'-GGAATTCATGATTTGGAAACGC-3' and 5'-CCCCAATTGCAATGAAATACTCTTT-3. The resulting amplicon was digested with EcoR1 and Mfe1 and ligated into the EcoR1 site of pEGFP-N1 and pDsRed2-N1 (BD Biosciences Clonetech). The mammalian expression vector pNecl-3-myc was constructed as follows: the cDNA DKFZp761G128 was used as a template for PCR amplification with the primers 5'-GGAATTCATGATTTGGAAACGC-3' and 5'-CCCCAATTGCAATGAAATACTCTTT-3. The resulting amplicon was digested with EcoR1 and Mfe1 and ligated into the EcoR1 site of pcDNA3.1/myc-His (Invitrogen). The mammalian expression vector pFc-Necl-3 was constructed as follows: the plasmid pNecl-3-myc was used as a template for PCR amplification with the primers 5'-AGGTCTATATAAGC-3' and 5'-GGGGTACCAGGGCCATTCTGGCC-3. The resulting amplicon was digested with Nhe1 and Kpn1 and ligated into the plasmid pIgplus (kindly provided by Patrick Doherty) digested with the same enzymes. The insect cell expression vector pMT-Fc-Necl-3 was constructed as follows: the pFc-Necl-3 vector was digested with Nhe1 and Apa1 and the resulting insert was ligated into the plasmid pMT (Invitrogen), digested with Spe1 and Apa1. The insect cell expression vector pMT-Necl-3-GFP and pMT-Necl-3-DsRed2 were constructed as follows: the pNecl-3-GFP and pNecl-3-DsRed2 vectors were digested with EcoR1 and Not1 and the resulting inserts were ligated into the plasmid pMT (Invitrogen), digested with the same enzymes. The bacterial expression vector pQE-Necl-3_(aa169-367) _was constructed as follows: the plasmid pNecl-3-myc was used as a template for PCR amplification with the primers 5'-AGGTCTATATAAGC-3' and 5'-GGGGTACCAGGGCCATTCTGGCC-3. The resulting amplicon was digested with Dra1 and Kpn1 and ligated into the blunted BamH1 and Kpn1 sites of the plasmid pQE-30 (Qiagen). The insect cell expression vectors pMT-Necl-1-GFP was constructed as follows: the cDNA IMAGE:5199627 (accession BC033819) was used as a template for PCR amplification with the primers 5'-CCCCAATTGATGGGGGCCCCAGCCGC-3' and 5'-CCCCAGATCTAAGATGAAATATTCCTTCTTGTCGTCCCC-3. The resulting amplicon was digested with Bgl2 and Mfe1 and ligated into the EcoR1 and BamH1 sites of pMT-Necl-3-GFP. The mammalian expression vector pNecl-2-GFP was constructed as follows: the cDNA IMAGE:4701395 (accession BC035930) was used as a template for PCR amplification with the primers 5'-GGAATTCATGGCGAGTGTAGTG-3' and 5'-CCCCAATTGCGATGAAGTACTCTTT-3. The resulting amplicon was digested with EcoR1 and Mfe1 and ligated into the EcoR1 site of pEGFP-N1 (BD Biosciences Clonetech). The insect vector pMT-Necl-2-GFP was constructed as follows: the pNecl-2-GFP vector was digested with EcoR1 and Not1 and the resulting inserts were ligated into the plasmid pMT (Invitrogen), digested with the same enzymes. The insect vector pMT-ALCAM-GFP was constructed as follows: the cDNA DKFZp667I089 (accession AL833702) was used as a template for PCR amplification with the primers 5'-CCCGAATTCATGGAATCCAAGGGGGCC-3' and 5'-CCCGGATCCAAGGCTTCAGTTTTGTGATTGTTTTCT-3. The resulting amplicon was digested with EcoR1 and BamH1 and ligated into the EcoR1 and BamH1 sites of pMT-Necl-3-GFP. The insect vector pMT-Necl-4-GFP, will be described in a manuscript in preparation (details are available on request).

### Cell transfection and aggregation assays

S2 Drosophila cells were maintained in Schneider's Drosophila Medium (Gibco) supplemented with 10% FCS. They were transfected with Lipofectamine 2000 (Invitrogen) in 6-well culture plates according to the manufacturer's recommendations. 24 hours after transfection the medium was changed and 0.5 mM CuSO_4 _was added for another 24 hours. For aggregation assays, relevant cells were mixed after the medium change, induced with CuSO_4 _and gently shaken for several hours to allow for plasmid transcription, protein accumulation and aggregate formation.

Hela cells were maintained in DMEM 10% FCS and transfected with Fugene (Roche) according to the manufacturer's recommendations.

### Protein expression

The E. coli strain M15 was transformed with the bacterial expression vector pQE-Necl-3_(aa169-367), _and used to express the corresponding Necl-3 fragment. Bacteria were grown exponentially, IPTG was added to 1 mM, and the cells were allowed to grow for another 4 h at 37°C. Cells were then harvested and lysed. The cleared supernatants were loaded under denaturing conditions onto Ni^2+^-nitrilotriacetic acid agarose columns (Qiagen), according to the manufacturer's recommendations. The eluted proteins were then extensively dialyzed against PBS and used for immunization.

The Fc-Necl-3 fusion protein was prepared as follows: the insect cell expression vector pMT-Fc-Necl-3 was co-transfected in S2 cells along with the plasmid pCoBlast that carries a blasticidin S resistance gene (Invitrogen). Stable clones were selected with blasticidin S according to the manufacturer's recommendations. Of these, high expressers of the Fc-Necl-3 fusion were selected. One such clone was expanded and grown in a 150 ml flat bottom flask and induced with 0.5 mM CuSO_4_. After 4 days, the conditioned culture medium was loaded onto a Protein A Sepharose CL-4B columns (Amersham Biosciences), and the Fc-Necl-3 fusion protein was recovered according to the manufacturer's recommendations.

### Antibody preparation

The Necl-3_(aa169-367) _fragment produced in E. coli was used by a commercial producer (Eurogentech, Belgium) to immunize two rabbits. The same Necl-3_(aa169-367) _fragment was coupled to NHS activated Sepharose 4 Fast Flow (Amersham Biosciences) according to the manufacturer's recommendations, and used for affinity purification of the anti Necl-3 antibodies from the rabbit sera. The antibody-containing fractions were then loaded onto a Protein A Sepharose CL-4B column (Amersham Biosciences). The beads were washed with 10 mM Tris pH 7.5, then 10 mM Tris pH 7.5, NaCl: 500 mM, and were then rocked for 2 hours at 4C as a suspension in 200 ml of 10 mM Tris pH 7.5, NaCl: 500 mM, in order to select only the antibodies with a very slow off-rate. The beads were then washed again with 10 mM Tris pH 7.5, and then 10 mM Tris pH 7.5, NaCl: 500 mM. Finally the IgGs were eluted according to the manufacturer's recommendations.

### Western blotting

Crude lysates from transfected S2 cells, or rat tissues were heated and sonicated in Laemmli sample buffer. Proteins were separated on a 12% SDS-polyacrylamide gels, and transferred to nitrocellulose membranes. These were probed with the appropriate antibodies and revealed by chemiluminescence (ECL, Amersham).

### Pull-down assays

S2 cells were transfected with the pMT-Necl-2-GFP plasmid in 6-well culture plates. After 48 hours the cells were washed in PBS and lysed in 400 μl RIPA buffer (Tris-HCl: 50 mM, pH 7.4, NP-40: 1%, NaCl: 150 mM, EDTA: 1 mM, and protease inhibitors). 100 μl of the resulting lysate was pre-cleared for 30 min at 4°C with 25 μl (bed volume) of Protein A Sepharose CL-4B resin. After pre-clearing, the lysates was rocked 45 min at 4°C with the Fc-Necl-3 fusion protein immobilized on Protein A Sepharose CL-4B resin. The beads were then washed 3 times with 500 μl RIPA buffer, and boiled in 1× Laemmli sample buffer for western blot analysis.

### Adhesion assays

Petri adhesion assays were done as described by Milner et al. [25]. Briefly, the indicated proteins at a concentration of 15 ng/μl were coated for 1 h at 37°C on a bacteriological grade Petri dish, after which unbound proteins were washed away with PBS. P1 rat cortices were dissected in ice cold HBSS, meninges were removed, and the tissue was minced with a fire-polished Pasteur pipette. A single cell suspension was prepared by passing the homogenate through a nylon mesh, and the cells were suspended in 10 ml Neurobasal medium (Gibco/Invitrogen) supplemented with 10% FCS and antibiotics. They were then spun for 5 min at 1500 g and resuspended at a concentration of 10^6 ^per ml. 50 μl of this suspension was added to the coated dish for 40 min at 37°C. Unbound cells were washed away by rinsing and gentle shaking in the same medium, and the bound cells were incubated for 24 h in fresh medium before imaging. Quantification was done manually on pictures taken with a microscope-mounted Leica digital camera at 200× magnification. Adhesion assay with Fc-Necl-3 added to the 14 DIV neonatal cortex cultures was done exactly as described [35], using 300 ng/100 μl of Fc-Necl-3 or human IgGs.

### Confocal and wide field immunofluorescence microscopy

Immunohistochemistry in the nervous system was done as follows: two-month-old rats were anaesthetized, their brains were rapidly excised and immersed for 5 min in isopentane pre-cooled to -20°C. Brains were then embedded in OCT medium (Miles Inc., Elkhart, IN) and cryo-sectioned at 12-μm. Sections were exposed for 10 min to -20°C absolute ethanol, rinsed in PBS and incubated for 30 min in PBS containing 2% BSA. Sections were then incubated for 2 h at 37°C in PBS, 0.5% BSA, containing the affinity-purified anti Necl-3 antibody (diluted 1:100) and the appropriate mouse monoclonal antibody. After washes in PBS, highly cross-adsorbed Alexa Fluor secondary antibodies (Molecular Probes) were used to detect the mouse and rabbit primary antibodies (with Alexa 488 and Alexa 594, respectively). Sections were then rinsed again in PBS and stained with Hoechst. Confocal laser scanning was performed on a Leica SP2 microscope (Leica, Germany) using 20 mW blue diode (405 nm), 30 mW ArKr (488 nm), 1 mW HeNe (594 nm) lasers and a 63× plan Apo oil immersion objective. Section planes were collected in steps through the entire cell thickness. Images of each field were merged using the Leica Confocal software. Z-section analysis was done with the Imaris software.

For immunofluorescence on cell cultures, Hela cells were grown on glass coverslips and transfected as indicated above. They were then fixed in PBS 3% paraformaldehyde for 5 min and quenched 5 min in PBS 50 mM NH_4_Cl. The cells were then blocked with PBS 10% FCS for 20 min, and incubated in PBS, 0.5% BSA, containing the affinity-purified anti Necl-3 antibody (diluted 1:200). After washes in PBS, highly cross-adsorbed Alexa Fluor secondary antibodies were as above. Coverslips were then rinsed again in PBS, and stained with Hoechst. Widefield microscopy was performed on a Zeiss AXIOZ1 microscope with a 63× oil immersion objective. Mouse mAbs were obtained from the following suppliers: DSHB (University of Iowa), neurofilament and Rip; Roche, GFAP and GFP; Serotec, CD11b; Chemicon, MBP and Upstate, Myc tag.

### Immunoelectron microscopy

Anesthetized rats were perfused with PBS containing 2% paraformaldehyde, 0,1% glutaraldheyde. Brain were dissected and put overnight in PBS with 2% paraformaldehyde, 25% sucrose for cryoprotection, then embedded in O.C.T. medium (Miles Inc., Elkhart, IN) and cryo-sectioned at 12 μm thickness. Sections were put on superfrost slides (Menzel GmbH, Braunschweig, Germany) and incubated for 30 min with PBS plus 0,1 % BSA (New England Biolabs, USA). Sections were incubated overnight in a humid chamber at 4°C with 40 μl of anti-Necl-3 antibody diluted (1:50) in PBS plus 0,1% BSA. They were then washed 5 times for 20 min with PBS plus 0,1 % BSA and incubated in a humid chamber with 40 μl of anti-rabbit IgG FAB fluoronanogold Alexa 488 (Nanoprobes Inc., Yaphank, NY, USA) diluted (1:10) in PBS (0,1% BSA, 0,1% FSK gelatine) for 2 h at room temperature. Sections were first washed with PBS plus 0.1% BSA (3 times 20 min) and then 3 times with 0,1 M sodium cacodylate (pH 7.2). After fixation with 2% glutaraldheyde in 0,1 M sodium cacodylate (30 min at room temperature) specimen were further washed with 0,1 M sodium cacodylate and distilled water. Gold enhancement of the nanogold particles was performed for 4 min according to the manufacturers' recommendations (Nanoprobes, Inc.). After enhancement, sections were washed 4 times with water and 3 times 10 min with 0,1 M sodium cacodylate. Sections were post-fixed in 0,1 M OsO_4 _for 1 h, washed 3 times 5 min with 0,1 M sodium cacodylate and 5 times 5 min with water and stained with uranyl acetate. Finally sections were dehydrated and embedded in resin (Agar 100 Resin, Agar Scientific, UK). Sections of 70 nm were collected on 200 slot grids, counterstained with half saturated uranyl acetate then 2.5 % lead citrate and analyzed using either a TEM Philips 410 or a Tecnai G2 electron microscope.

### Real time PCR analysis

Real time PCR analysis were prepared with SYBR green (Molecular Probes) as described [36] on an iCycler iQ Bio-Rad station, with quantitation, melt curve, and PCR efficiency analysis derived by the station's software. RNA purification and cDNA synthesis were prepared as described [17]. The primers used for amplifying Necl-3 were 5'-TGACCATGCTCTCATAGG-3' and 5'-TGCCAGATATCGACCAAG-3', those for GAPDH were 5'-TGATTCTACCCACGGC-3' and 5'-TGATGGGTTTCCCATTGATGA-3'.

## Authors' contributions

FP performed the largest part of the work, AG helped for DNA cloning and aggregation assays, CB assisted and supervised the confocal microscopy, MB is the lab PI and helped for the manuscript writing, and VO initiated and supervised the work and wrote the manuscript. All authors have read and approved the final version of the manuscript.
